# A NEW ANALYTICAL METHOD FOR RISK FACTOR IDENTIFICATION IN ANALYZING LONGITUDINAL POSTFRACTURE RECOVERY OUTCOMES

**DOI:** 10.1093/geroni/igae098.2086

**Published:** 2024-12-31

**Authors:** Chixiang Chen, Michelle Shardell, Jason Falvey

**Affiliations:** University of Maryland School of Medicine, Baltimore, Maryland, United States; University of Maryland School of Medicine, Baltimore, Maryland, United States; University of Maryland School of Medicine, Baltimore, Maryland, United States

## Abstract

Hip fracture is a highly disabling event experienced disproportionately by older adults with Alzheimer’s disease or related dementias (ADRD). Older adults with ADRD are up to three times more likely than cognitively intact older adults to sustain a hip fracture, have worse functional outcomes, and spend more than 50 fewer days at home (DAH) in the year after fracture. Given the significant consequences of hip fracture among older adults, improving recovery for those with ADRD is a crucial national priority. However, the population of patients with ADRD is highly heterogeneous, hindering our ability to risk stratify patients and focus interventions on those at highest risk and with greatest likelihood of benefit. We develop a new data science approach to unbiasedly detect high-dimensional patient-level risk factors associated with less DAH, while existing toolboxes may fail to address potential biases from unmeasured hospital-level confounders, informative hospital size, and loss to follow-up in analyzing Medicare claims. Analysis of 16562 Medicare beneficiaries with ADRD, aged 65 or above, and experienced a hip fracture between 2017 and 2019 demonstrated that older age, longer length of stay in the hospital, heavier comorbidity burden, and less days at home before fracture are significantly associated less days spent at home in the 12-month period after hip fracture (p<.05), while existing methods fail to detect some effects. Findings demonstrate that the proposed method can overcome bias in using Medicare data and can lead to findings that may inform target for intervention to promote healthy aging.

